# Efficacy of contrast-enhanced ultrasound for diagnosis of cesarean scar pregnancy type

**DOI:** 10.1097/MD.0000000000017741

**Published:** 2019-11-01

**Authors:** Yun Wu, Liuying Zhou, Lin Chen, Qian Zhou, Tao Zeng

**Affiliations:** Department of ultrasound, Chengdu Women's and Children's Central Hospital, 1617th Riyue Road, Qingyang District, Chengdu 610000, Sichuan province, China.

**Keywords:** cesarean scar pregnancy, contrast-enhanced ultrasound, transvaginal ultrasound

## Abstract

**Objectives:**

We compared the clinical efficacy of contrast-enhanced ultrasound (CEUS) to transvaginal ultrasound (TVS) for diagnosing cesarean scar pregnancy (CSP).

**Methods:**

A total of 485 cases of suspected CSP were recruited from January 2017 to March 2018. All received TVS and CEUS by two sonologists blinded to diagnosis by the other. Diagnostic features of CSP that significantly differed between modalities by univariate analysis (*P* < .05) were included in a logistic regression model. The sensitivity, specificity, positive likelihood ratio (+LR), negative likelihood ratio (−LR), and accuracy (ACC) of CSP diagnosis by TVS and CEUS were compared according to operational and pathological outcomes as the reference standard.

**Results:**

There were 220 CSP cases (including 85 cases of type I, 93 of type II, and 42 of type III). The sensitivities of CEUS for detection of types I − III CSP were 94.1%, 92.5%, and 97.6%, respectively, and corresponding sensitivities of TVS were 82.4%, 80.6%, and 95.2%. Compared to TVS, CEUS yielded significantly better overall sensitivity (97.27% vs 88.18%), specificity (96.60% vs 75.47%), +LR (28.60 vs 3.59), −LR (0.03 vs 0.16), and diagnostic ACC (96.9% vs 81.23%) (all *P* < .001).

**Conclusions:**

CEUS is superior to TVS for detecting cesarean scar pregnancy and distinguishing among CSP types.

## 1 Introduction

Cesarean scar pregnancy (CSP) is a rare form of ectopic pregnancy in which the gestational sac is partially or completed implanted in the region of a cesarean section scar.^[1]^ With trophoblastic cell implantation and tissue erosion, the gestational sac can implant in the scar and/or myometrium, even penetrating the myometrium of the incision region.

In recent years, delivery by cesarean section has become more frequent in China.^[2]^ According to the World Health Organization (WHO), China ranks first among Asian countries with 46.2% of deliveries by cesarean section.^[3]^ Jurkovieh et al^[4]^ reported that the incidence of CSP in women with a history of cesarean section is as high as 0.15%.^[4]^ With the advent of the “one couple two children” policy, the incidence of CSP is increasing in China, and is now substantially higher than in other Asian countries. If CSP patients fail to receive timely diagnosis and treatment, life-threatening hemorrhage and uterine rupture may occur in severe cases.^[5]^ Therefore, termination of pregnancy in the first trimester is strongly recommended for the management of CSP.^[6]^

Early diagnosis and risk assessment of CSP can guide subsequent treatment decisions and reduce potential risks to the mother's health. Transvaginal ultrasound (TVS) is the most common modality for CSP diagnosis and one recent study reported a diagnostic sensitivity of 86.4%.^[7]^ To further improve the diagnostic accuracy of CSP, identify CSP type, and more accurately assess CSP risk, contrast-enhanced ultrasound (CEUS) is increasing applied in clinical practice. The main advantage of CEUS is the ability to image the CSP microcirculation. However, the efficacy of CEUS for diagnosis and assessment of CSP type, which determines the most appropriate treatment, has not been systematically compared to TVS. This study compared the clinical efficacy of CEUS to TVS for diagnosing CSP type.

## 2 Materials and methods

This was a prospective study conducted at the Ultrasound Department. From January 2017 to March 2018, about 13000 pregnant women with history of the cesarean section were examined at our hospital, a tertiary center for women and children's health. A total of 485 cases of early pregnancy with suspected CSP were identified during the study period (mean age, 31 years; range, 21–42 years). Inclusion criteria were positive serum β-human chorionic gonadotropin (β-HCG) level, gestational age less than 11 weeks, gestational substance located at or near the lower uterine segment, and a history of cesarean section in the lower uterine segment. Exclusion criteria were further reproductive plans, allergy to the contrast agent, and other contraindications for CEUS.

It is unknown whether the microbubbles of contrast agent pass through the placenta to impact the fetus, although this seems unlikely.^[8]^ Nonetheless, all pregnant women included had no future reproductive plans and wanted to terminate the pregnancy at the early stage.

All ultrasound examinations were conducted using a EPIQ7 system (Philips Ultrasound, Inc, Bothell, WA) equipped with 3 to 10 MHz convex probe (C10-3 v, Philips). Subjects were first examined by TVS and then by CEUS. Respective examinations were performed by two independent sonologists, each with fourteen years working experience. Both had been trained and performed examinations according to European Federation of Societies for Ultrasound in Medicine and Biology (EFSUMB) guidelines.^[8]^ Both sonologists were blind to the other's diagnosis. The surgical and histopathological outcomes were obtained in all cases and used as the reference standard for judging diagnostic accuracy.

### 2.1 TVS examination

The bladder was emptied before examination. The goals of the TVS scan were to determine the location, size, and shape of the gestational substance, uterus, adnexal area, and pelvic cavity, measure myometrium thickness in the incision region, and assess blood flow.

### 2.2 CEUS examination

**Figure 1:**
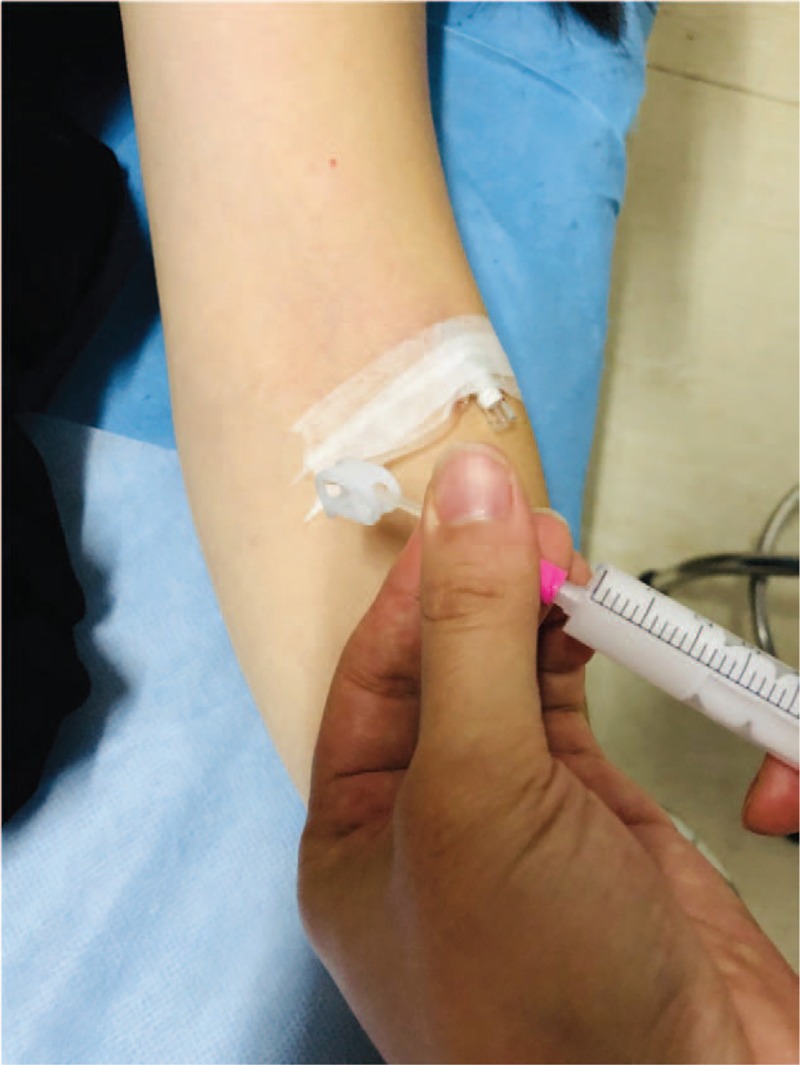
Injected through elbow vein.

Written informed consent was obtained from all patients before CEUS examination. Under the contrast mode, the mechanical index (MI) ranged between 0.05 and 0.07.^[9]^ SonoVue (Bracco International B.V, Amsterdam, The Netherlands) was used as the contrast agent in all cases. Briefly, 25 mg of contrast agent was dissolved in 5 ml of 0.9% sodium chloride and injected as an intravenous bolus (2.4 ml per subject) through the elbow vein, followed by a 5-ml normal saline flush (Fig. 1).^[8–10]^ Upon injection, the time key and dynamic storage key were pressed. Dynamic images of the regions of interest were recorded for a minimum of 2 minutes and stored on the machine's internal hard drive for off-line analysis. Regions of interest included the gestational substance (location, size, and shape), uterine incision region, myometrium, and microcirculation. According to the difference in peak enhancement between gestational substance and myometrium, enhancement was classified as hypo-enhancement, iso-enhancement, hyper-enhancement, or no-enhancement. The time of enhancement was classified as early, synchronous, or later.^[9]^

According to the Family Planning Group, Chinese Medical Society of Obstetrics and Gynecology Expert Consensus on Diagnosis and Treatment of Cesarean Section Scar Pregnancy, CSP was classified into 3 types. In this classification standard, the main sonographic features include location and shape of the gestational substance, myometrium thickness in the incision region, and blood flow characteristics. This classification method is useful for subsequent treatment guidance.^[11]^

### 2.3 Diagnostic standards for CSP type

**Figure 2:**
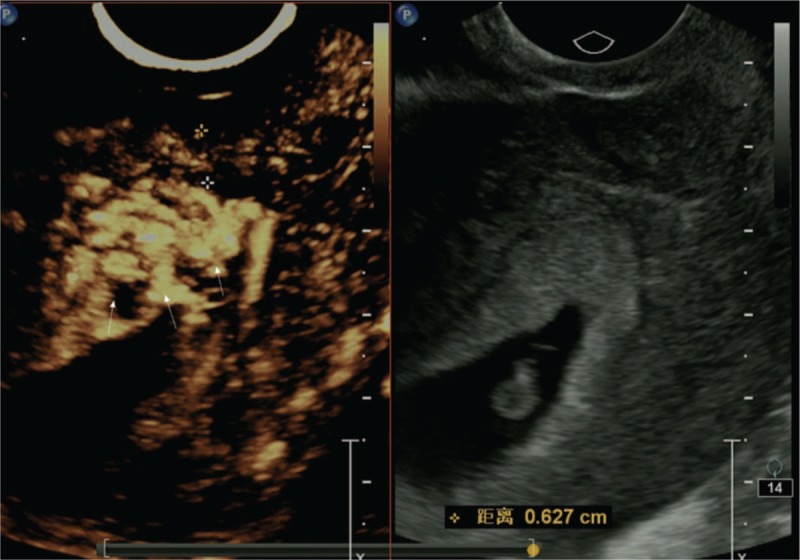
Type I.

Type I CSP (Fig. 2) is defined according to the following criteria^[11]^:

1. gestational substance partially located in the incision region of the uterus,
2. irregular gestational substance shape,
3. myometrium thickness in the incision region > 3 mm, and
4. blood flow observed in the gestational substance located within the incision region.

**Figure 3:**
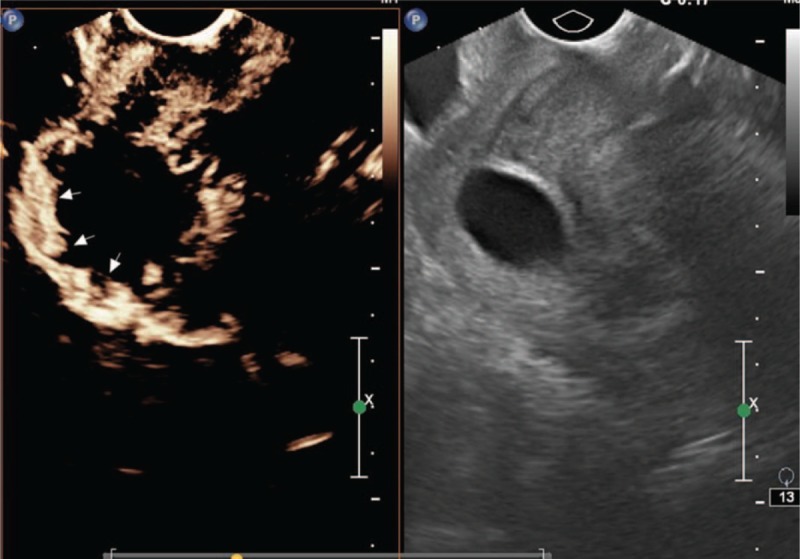
Type II.

Type II CSP (Fig. 3) is defined by the following^[11]^:

1. gestational substance partially located in the incision region of the uterus,
2. irregular gestational substance shape,
3. myometrium thickness in the incision region ≤ 3 mm, and
4. blood flow observed in the gestational substance located within the incision region.

**Figure 4:**
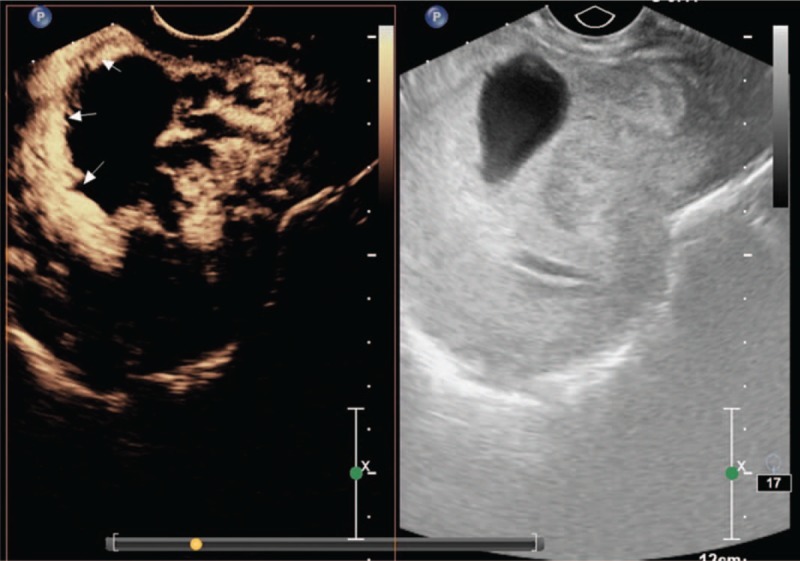
Type III.

Finally, type III CSP (Fig. 4) is defined by the following^[11]^:

1. gestational substance located completely within the incision region of the uterus,
2. gestational substance of irregular or regular shape,
3. myometrium thickness at the incision region ≤ 3 mm, and
4. blood flow observed in the gestational substance within the incision region.

**Figure 5:**
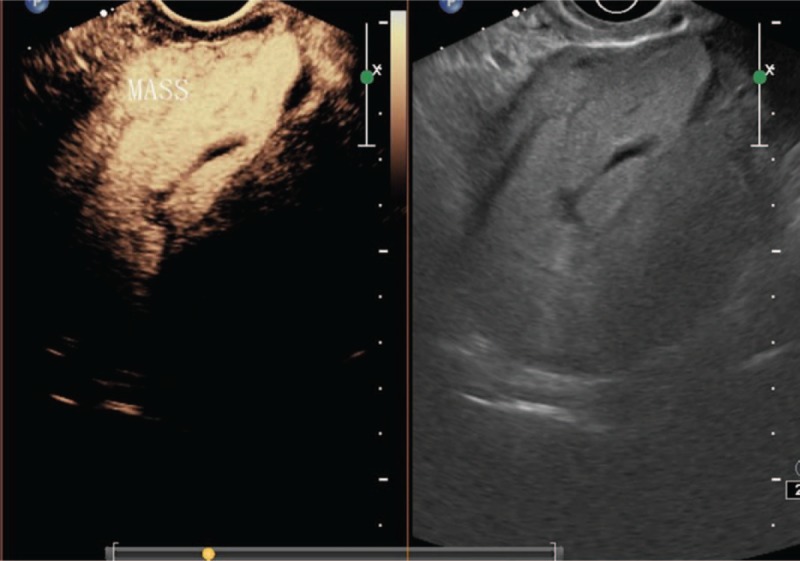
Type III (mass).

A special sonographic subtype was found among type III cases termed mass type (cystic and solid mixed echo or solid mass) (Fig. 5). The mass type was usually formed due to pregnancy residue and hemorrhage in the scar of the uterus after CSP abortion (such as after drug-induced abortion or after negative pressure suction).^[6,11]^

### 2.4 Data analysis

The required sample size was calculated and statistical tests performed using SPSS version 17.0 (SPSS Inc, Chicago, IL). A logistic regression model was constructed including all features differing significantly between CSP and non-CSP cases (*P* < .05, 2-tailed). The sensitivity, specificity, positive likelihood ratio (+LR), negative likelihood ratio (−LR), and ACC of CSP type diagnosis by TVS and CEUS were calculated based on surgical and pathological results as the reference standard. Group enumeration data were compared by Chi-square test and measurement data by X. A *P* < .05 (2-tailed) was considered statistically significant for all tests.

### 2.5 Ethical approval

The study was discussed and approved by the Ethics Committee of Chengdu Women's and Children's Central Hospital, and the ID: 2016-3-1759. All information of the study had been recorded by the Ethics Committee of Chengdu Women's and Children's Central Hospital. Written informed consent was obtained from all pregnant women before the examination.

## 3 Results

### 3.1 Surgical and pathological results (the reference standard)

Among the 485 cases of suspected CSP, 220 cases were confirmed by surgery and pathological analysis (including 85 cases of type I, 93 of type II, and 42 of type III), while 265 cases were diagnosed as non-CSP (including intrauterine pregnancy, abortion, and uncertain diagnosis). Patient clinical data and sonographic characteristics of CSP are summarized in Table 1. Among the sonographic characteristics recorded, the location and shape of the gestational substance (GS), myometrium thickness within the incision region, blood flow within the incision region, and the enhancement pattern differed significantly between CSP and non-CSP cases.

**Table 1:**
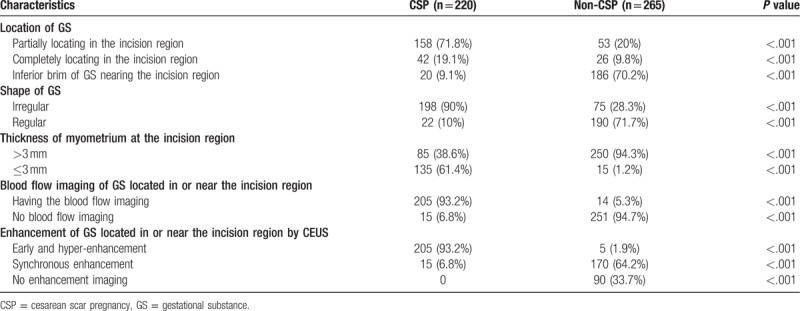
Patient clinical data and sonographic characteristics of cesarean scar pregnancy (CSP).

Factors differing significantly between CSP and non-CSP women were then included in a logistic regression model (Table 2). Factors significant for CSP diagnosis with high inter-observer agreement included gestational substance partially located within the incision region (95%CI: 15.894–48.359), gestational substance completely within the incision region (95%CI: 7.670–29.425), irregular shape of the gestational substance (95%CI: 0.026–0.073), thickness of the myometrium within the incision region (95%CI: 14.711–47.632), blood flow within this region (95%CI: 0.002–0.009), and earlier hyper-enhancement (95%CI: 1.476–6.535).

**Table 2:**
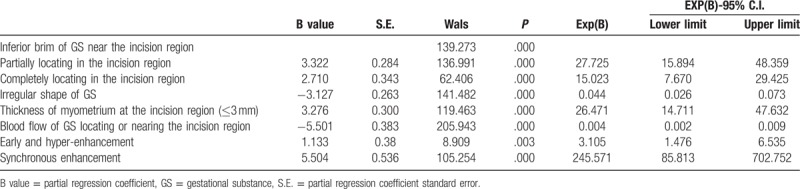
Logistic regression analysis of factors distinguishing CSP from non-CSP.

The sensitivity, specificity, +LR, −LR, and ACC were higher using CEUS than TVS (*P* < .001). All data are summarized in Tables 3 to 5.

**Table 3:**
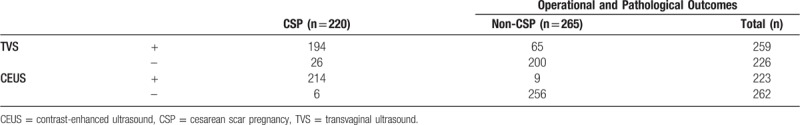
Diagnosis of CSP by TVS vs CEUS.

**Table 4:**
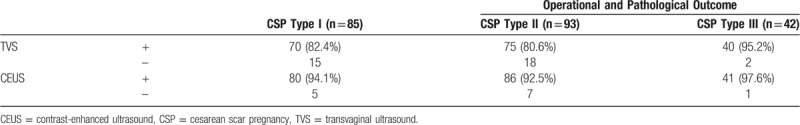
CSP type identification by TVS vs CEUS.

**Table 5:**
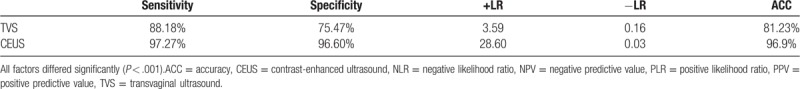
Comparison of CSP diagnostic efficacy between TVS and CEUS.

## 4 Discussion

Cesarean scar pregnancy is one of the long-term risks of cesarean delivery, and CSP frequency is on the rise in China due to the high incidence of cesarean section delivery and the recent 2-child policy.^[2]^ After cesarean section, tissue structure is weakened at the incision scar region, which is frequently located at the isthmus, due to the absence of normal endometrium and myometrium.^[12]^ The isthmus of the uterus has an abundant blood supply, however. If the fertilized egg is implanted in the incision region and trophoblast cells implant directly into the myometrium with concomitant erosion of large blood vessels, life-threatening bleeding can occur.^[12]^ Therefore, early diagnosis and assessment of CSP are critical.

Compare with other imaging technologies, ultrasound is more convenience, cheaper, safer for patients ^[13]^. Jurkovie et al^[4]^ proposed TVS with color Doppler as the first choice for the diagnosis of ectopic pregnancy in the lower part of the uterus. In this protocol, the location, size, and shape of the gestational sac, the spatial relationship between the gestational sac and incision region, and blood flow surrounding the gestational sac would be initially evaluated by TVS.^[4]^ However, sonographic imaging of CSP is complex and TVS may lead to misdiagnosis in some cases. For example, blood flow between the gestational sac and the incision region may be undetectable due to weak signal. In addition, the range of gestational sac implantation cannot be accurately judged by TVS.

Alternatively, CEUS can compensate for these deficiencies by enhanced imaging of the microcirculation. In normal pregnancy, the spiral artery and vein of the uterus open end in the villus space. In CSP, however, the arcuate artery directly opens in the villus space with significantly increased pressure due to vessel erosion, and contrast agent facilitates real-time dynamic display of high-pressure microcirculation in CSP.

Among the sonographic characteristics recorded, the location and shape of the gestational substance (GS), myometrium thickness within the incision region, blood flow within the incision region, and the enhancement pattern differed significantly between CSP and non-CSP cases (Table 1). In this study, CSP was classified into 3 types according to location and shape of the gestational substance, myometrium thickness in the incision region, and blood flow pattern in the incision region.^[11]^ Compared to the previous 2-type classification scheme (type I, endogenic type; type II, exogenic type),^[14,15]^ classification into 3 types is more precise and provides superior treatment guidance.^[11]^ Each of the CEUS metrics differed significantly between CSP and non-CSP cases, thereby providing accurate differential diagnosis (Tables 1 and 2). Furthermore, the enhancement pattern (earlier enhancement and hyper-enhancement) on CEUS can more clearly reveal CSP blood flow, while non-CSP cases (abortion or implantation near but not within the incision region) showed no blood flow between the gestational substance and the incision region.

Analysis of many hundreds of cases using TVS also yielded high sensitivity (88.18%), high specificity (75.47%), and low −LR (0.16) for distinguishing CSP from non-CSP. Thus, TVS can also exclude most obvious non-CSP cases and diagnose most classical CSP cases. However, there were many cases that could not be accurately diagnosed by TVS, such as those in which the gestational sac was near the cesarean scar but not implanted in the scar as well as cases with weak blood flow. In addition, TVS was less sensitive than CEUS for identification of both type I (82.4%) and type II (80.6%) CSP. The reasons for this reduced accuracy can be summarized as follows:

1. when the lesion was close to the incision area and the blood flow signal was hard to detect, CSP could not be definitely excluded or accurately diagnosed,
2. mass-like CSP is prone to misdiagnosis and missed diagnosis by TVS if there is a lot of accumulated blood and no obvious blood flow signal, and
3. TVS cannot distinguish the boundary of the implantation. However, through enhancement of the microcirculation by CEUS, the scope of the villi bed can be precisely delineated. The diagnostic sensitivity of CEUS was markedly higher than TVS for both CSP type I (94.1%) and type II (92.5%). The specificity, +LR, −LR, and ACC were also superior. Indeed, most of the false positive cases by TVS could be excluded by CEUS.

However, there were certain limitations to CEUS as well. It is unknown whether the microbubbles of contrast agent pass through the placenta to impact the fetus (although this seems unlikely ^[8]^), so we did not assess CEUS for women planning to maintain the pregnancy. In the future, we hope to investigate the clinical utility of CEUS on such women.

In this study, CEUS was applied for cases of suspected CSP to diagnosis and identify CSP type and assess the risks. These subtypes of CSP are distinguished mainly by the depth of implantation and have distinct levels of risk and treatment requirements. For example, the risk associated with type III is higher than that of type I, so treatment is chosen based on this elevated risk. According to accurate type and risk assessment, the clinician can choose the most appropriate treatment, such as chemical treatment, uterine artery embolism (UAE), or surgical treatment, such as hysterectomy, gestation removal and surgical repair of uterine scar by laparotomy, laparoscopy, or laparoscopy combined with hysteroscopy, and hysterectomy by vaginal. The choice of surgical treatment is based on classification, the risk of bleeding and fertility requirements. In addition, pretreatments such as UAE can be performed before surgery if there is a high risk of bleeding.^[11]^

## 5 Conclusions

Contrast-enhanced ultrasound (CEUS) demonstrated superior diagnostic and classification efficacy for CSP compared to TVS due to more precise delineation of GS location, range, depth, and microcirculation pattern.

## Acknowledgments

Thanks to the Chengdu Women and Children's Center Hospital for support.

## Author contributions

**Conceptualization:** Yun Wu.

**Formal analysis:** Yun Wu.

**Investigation:** Yun Wu, Lin Chen, Qian Zhou, Tao Zeng.

**Project administration:** Liuying Zhou.

**Resources:** Liuying Zhou, Lin Chen.

**Writing - Original Draft:** Yun Wu.
